# The association between primary care appointment lengths and opioid prescribing for common pain conditions

**DOI:** 10.1186/s12913-024-11215-5

**Published:** 2024-07-02

**Authors:** John C. Matulis, Kristi Swanson, Rozalina McCoy

**Affiliations:** 1 Division of Community Internal Medicine, Geriatrics and Palliative Care, Department of Medicine, Mayo Clinic, Rochester, MN USA; 2 Kern Center for the Science of Health Care Delivery, Mayo Clinic Robert D. and Patricia E, Rochester, MN USA; 3 Division of Endocrinology, Diabetes, & Nutrition, Department of Medicine, University of Maryland School of Medicine, Baltimore, MD USA; 4 University of Maryland Institute for Health Computing, Bethesda, MD USA; 5 Division of Gerontology, Department of Epidemiology and Public Health, University of Maryland School of Medicine, Baltimore, MD USA; 6 Department of Health Policy and Management, University of Maryland School of Public Health, College Park, MD USA

**Keywords:** Schedules and appointments, Primary care, Practice patterns, Opioids, Quality, Safety of healthcare, Internal medicine, Family medicine

## Abstract

**Background:**

While brief duration primary care appointments may improve access, they also limit the time clinicians spend evaluating painful conditions. This study aimed to evaluate whether 15-minute primary care appointments resulted in higher rates of opioid prescribing when compared to ≥ 30-minute appointments.

**Methods:**

We performed a retrospective cohort study using electronic health record (EHR), pharmacy, and administrative scheduling data from five primary care practices in Minnesota. Adult patients seen for acute Evaluation & Management visits between 10/1/2015 and 9/30/2017 scheduled for 15-minute appointments were propensity score matched to those scheduled for ≥ 30-minutes. Sub-groups were analyzed to include patients with acute and chronic pain conditions and prior opioid exposure. Multivariate logistic regression was performed to examine the effects of appointment length on the likelihood of an opioid being prescribed, adjusting for covariates including ethnicity, race, sex, marital status, and prior ED visits and hospitalizations for all conditions.

**Results:**

We identified 45,471 eligible acute primary care visits during the study period with 2.7% (*N* = 1233) of the visits scheduled for 15 min and 98.2% (*N* = 44,238) scheduled for 30 min or longer. Rates of opioid prescribing were significantly lower for opioid naive patients with acute pain scheduled in 15-minute appointments when compared to appointments of 30 min of longer (OR 0.55, 95% CI 0.35–0.84). There were no significant differences in opioid prescribing among other sub-groups.

**Conclusions:**

For selected indications and for selected patients, shorter duration appointments may not result in greater rates of opioid prescribing for common painful conditions.

**Supplementary Information:**

The online version contains supplementary material available at 10.1186/s12913-024-11215-5.

## Background

Of the 50,000 opioid-related overdose deaths reported in 2017 [1], 40% (approximately 20,000 deaths) involved prescribed opioids [2]. By 2022, annual opioid-related overdoses have increased to over 100,000 deaths per year [3]. In addition to this staggering death toll, approximately 11.5 million people misuse prescription opioids annually, leading to substantial social and medical harms as well as risk of future addiction and overdose [4]. As approximately half of all opioid prescriptions are issued in primary care settings [5], responsible opioid stewardship in this setting is critical to managing this public health crisis.

Despite efforts to promote standardized practices around opioid prescribing [6] and the release of national guidelines [7] on appropriate prescribing, there is substantial variation in opioid prescribing practices in primary care [8–12]. Several clinician level factors have been proposed to explain this variation. Training in pain management and substance use disorders among primary care clinicians is variable [13] and generally considered inadequate [14]. Other clinician specific factors include perceived medical contraindications to non-opioid alternatives, the inability of clinicians to address difficult social circumstances which may limit non-opioid treatment options, and a lack of time to perform shared decision-making conversations around opioid prescribing [15]. However, less is known about how specific organizational factors may impact primary care provider opioid prescribing. While prior study found that primary care appointments scheduled later in the day were associated with increased rates of opioid prescribing [16, 17], less is known about how other organizational factors like care team support, clinical resources and practice structure may impact opioid prescribing.

We hypothesize that another organizational factor that may influence opioid prescribing in the primary care setting is the scheduled duration of an acute appointment. While brief duration appointment lengths may help address gaps in access to care for painful conditions by increasing the total number of available appointments, they may have unintended consequences on care delivery. When there is inadequate time available to support nuanced and patient centered conversations around different pain management modalities and the risks and benefits of opioid prescribing, clinicians may default to initiating or continuing opioid therapy, potentially deferring difficult conversations, checking the state prescription drug monitoring program, or completing an opioid risk screening tool [18]. A more detailed understanding of how time pressures may impact opioid prescribing during acute primary care visits may help inform and guide interventions to improve opioid stewardship in the primary care setting.

## Methods

### Study design

We performed a retrospective cohort study using a combination of institutional billing, scheduling, and pharmacy data from Mayo Clinic, Rochester, MN to compare rates of opioid prescribing as a function of appointment lengths during evaluation and management (E&M) visits for painful conditions rendered within the Mayo Clinic Department of Employee and Community Health (ECH) from 10/1/2015 to 9/30/2017. This study was approved by the Mayo Clinic Internal Review Board (19-001641) and is reported in adherence to STROBE reporting guidelines [19].

### Setting

Mayo Clinic is an integrated healthcare delivery system serving local, regional, national, and international patients with a clinical and academic hub in Rochester, MN. Mayo Clinic’s outpatient department of Employee and Community Health (ECH) provides longitudinal primary care to approximately 120,000 local area residents, including Mayo Clinic employees and their dependents (comprising approximately 50% of the ECH patient population) across five locations in Olmsted County, Minnesota, representing urban, suburban, and rural care settings. The ECH practice is integrated with the tertiary care practice of Mayo Clinic Rochester, and ECH patients do have access to specialty and sub-specialty pain, procedural and addiction resources. The practice employs approximately 55 Internal Medicine physicians, 12 Internal Medicine Nurse Practitioners or Physician’s Assistants, 60 Family Medicine physicians, 35 Family Medicine Nurse Practitioners or Physician’s Assistants, 100 Internal Medicine residents and 25 Family Medicine residents.

### Study population

We identified adult patients (aged ≥ 18 years) presenting for acute outpatient office (“index”) visits in the Community Internal Medicine (CIM) and Family Medicine (FM) practices of Mayo Clinic, Rochester between 10/1/2015 and 9/30/2017. Included patients were empaneled to a Mayo Clinic primary care provider for at least one year prior to the index visit (to allow for ascertainment of baseline characteristics and risk factors) and for 30 days after (to allow for complete outcome ascertainment). Index visits were identified using Current Procedural Terminology (CPT) codes for office or other outpatient Evaluation & Management (E&M) visits (99,201–99,215 and 99,241–99,245).

To identify visits for a new, painful chief complaint, we excluded visits preceded by another similarly defined E&M visit in primary care within the preceding two weeks. Visits only for preventive services and visits with non-primary care providers (e.g., Registered Nurse, Licensed Practical Nurse, Certified Nurse Specialist, dietician, social worker, etc.) were excluded from analysis. Patients who did not provide authorization for their data to be used for research were excluded in accordance with Minnesota state law [20]. 

### Identification of visits for acute painful conditions

The primary diagnosis from administrative billing data was used to obtain the reason for the visit and was filtered to include common painful conditions routinely managed in primary care, identified through literature review [21–25], authors’ clinical experience, and feedback from subject matter experts. These conditions were identified using ICD-9 and ICD-10 diagnosis codes, as well as the Clinical Classification Software Refined categories developed by the Healthcare Cost and Utilization Project [26]. Both forward mapping (ICD-9 to ICD-10) and backward mapping (ICD-10 to ICD-9) was performed using the General Equivalence Mappings [27] to enumerate all relevant diagnosis codes of interest for the study period examined. The ICD-10 codes for the full list of selected conditions are presented in Appendix A.

Common painful conditions were categorized as acute or chronic by author review (RGM and JCM). In the event of disagreement, a consensus process was used to categorize the diagnosis codes as either acute or chronic. If the authors determined a diagnosis code could plausibly be either acute or chronic, a look back period of 12 months was examined, excluding the 30 days directly prior to the index visit (because that would have disqualified this visit from being included in the dataset). If another visit with a similar indication was identified during this time, the condition was considered chronic, otherwise it was considered acute.

### Independent variables

The main exposure of interest was the scheduled appointment length of the index visit. Appointment lengths were ascertained from administrative scheduling data and categorized as 15 min (brief duration) versus 30 min or longer (long duration). The length of time allotted for a particular appointment is usually determined by centralized scheduling staff members using standardized scheduling templates (Appendix B) based on the patient’s stated health concern and patient characteristics; however, schedulers and primary care physicians may substitute their own judgement and schedule patients into longer or briefer appointment slots as deemed appropriate to meet patient needs.

Covariates of interest included patient and clinician level factors. Patient characteristics extracted from the EHR included age, ethnicity, race, sex, marital status, and geographic location. Limited English proficiency was identified from registration data. Using ICD-9 and ICD-10 diagnosis codes from billing data, the Deyo adaptation of the Charlson comorbidity index was calculated with severity weighting incorporated [28–30]. Provider level covariates included primary care specialty (Internal Medicine vs. Family Medicine), Clinician type (NP/PA, physician, or resident).

### Outcomes

The primary outcome was whether an opioid medication was prescribed on the same day as the index visit. Data on opioid prescribing was ascertained from electronic order entry within the EHR and linked to the study population based on the ordering date of the prescription.

### Determination of prior opioid exposure

Opioid use in the 6 months prior to the index visit was obtained from EHR prescription data to determine whether a patient was opioid naïve (no prior opioid prescription issued in the preceding 6 months) or had prior opioid exposure (any prior opioid prescription issued within the past 6 months).

### Statistical analyses

Patient and visit characteristics were summarized for all eligible visits, which were subset into four mutually exclusive categories: acute pain–opioid naïve patient, acute pain–prior opioid use patient, chronic pain–opioid naïve patient, chronic pain– prior opioid use patient. Patient characteristics were summarized in aggregate and compared across differing appointment lengths to assess for significant associations using Chi-Square tests for categorical variables and Kruskal-Wallis tests for continuous variables.

The distribution of appointment lengths was summarized across the four subgroups. It was anticipated that the length of scheduled appointments would depend on certain patient and system level factors (i.e., medical complexity, social support, triage criteria, etc.). Therefore, propensity score methods by means of covariate adjustment were used to account for potential selection bias in the exposure of interest. Full details regarding the propensity score matching approach used are provided in Appendix C.

Multivariate logistic regression was performed to examine the effects of appointment length on the likelihood of an opioid being prescribed, while adjusting for other covariates, including ethnicity, race, sex, marital status, and prior ED visits and hospitalizations. A separate model was computed and reported for each of the four subgroups. The calculated propensity score for each observation was included as a separate covariate in each of the models. Odds ratio (OR) estimates were computed, along with corresponding 95% confidence intervals (CI) and *p*-values, for each model. All data management and analyses were performed using SAS 9.4 (SAS Institute Inc. Cary, NC).

## Results

We identified 45,471 eligible acute care visits to primary care during the study period. Both brief and longer duration appointments were evenly distributed between the practice areas **(**Table 1**)**, with 2.7% (*N* = 1233) of the visits being brief duration (15 min) and 98.2% (*N* = 44,238) scheduled for 30 min of longer. Baseline numbers of appointments based on pain indication (acute vs. chronic) and prior opiate exposure (naïve vs. chronic) by appointment duration are included in Supplemental Table 1 while rates of opiate prescribing per selected cohort are included in Table 2.

**Table 1:** Baseline characteristics of the study population

		N (%) (*N* = 45,500)
**Age (mean; Std.)**		54.7 (17.98)
**Practice Area**		
	Family Medicine	23,552 (51.8%)
	Internal Medicine	21,948 (48.2%)
**Provider Type**		
	Physician	24,652 (54.2%)
	NP/PA	13,967 (30.7%)
	Resident	6,881 (15.1%)
**Patient’s Preferred Language**		
	English	43,714 (96.1%)
	Non-English	1,762 (3.9%)
	Unknown	24 (0.1%)
**Charlson Index**		
	0	24,294 (53.4%)
	1	8,601 (18.9%)
	2	4,464 (9.8%)
	3	2,754 (6.1%)
	4	1,662 (3.7%)
	5 or more	3,725 (8.2%)
**Ethnicity**		
	Hispanic	913 (2.0%)
	Not Hispanic	43,353 (95.3%)
	Unknown	1,234 (2.7%)
**Race**		
	Black	1,652 (3.6%)
	Asian	1,200 (2.6%)
	Other/Unknown	1,900 (4.2%)
	White	40,748 (89.6%)
**Sex**		
	Female	28,354 (62.3%)
	Male	17,146 (37.7%)
**Marital Status**		
	Married or Partnered	29,504 (64.8%)
	Not Married or Unknown	15,996 (35.2%)

**Table 2:** Crude Rates of same-day opioid prescribing by sub-group

Patient Characteristics: Acute vs. chronic Pain and Opioid Naïve vs. Chronic Opioid exposure	Opioid Prescribed *N* = 3,246 (7.1%)	Opioid Not Prescribed *N* = 42,254 (92.9%
Acute Pain indication, Opioid Naïve	1,138 (4.2%)	25,976 (95.8%)
Acute Pain indication, Chronic Opioid exposure	659 (14.0%)	4,045 (86.0%)
Chronic Pain indication, Opioid Naïve	412 (4.4%)	8,864 (95.6%)
Chronic Pain indication, Chronic Opioid exposure	1,037 (23.5%)	3,369 (76.5%)

**Fig. 1:**
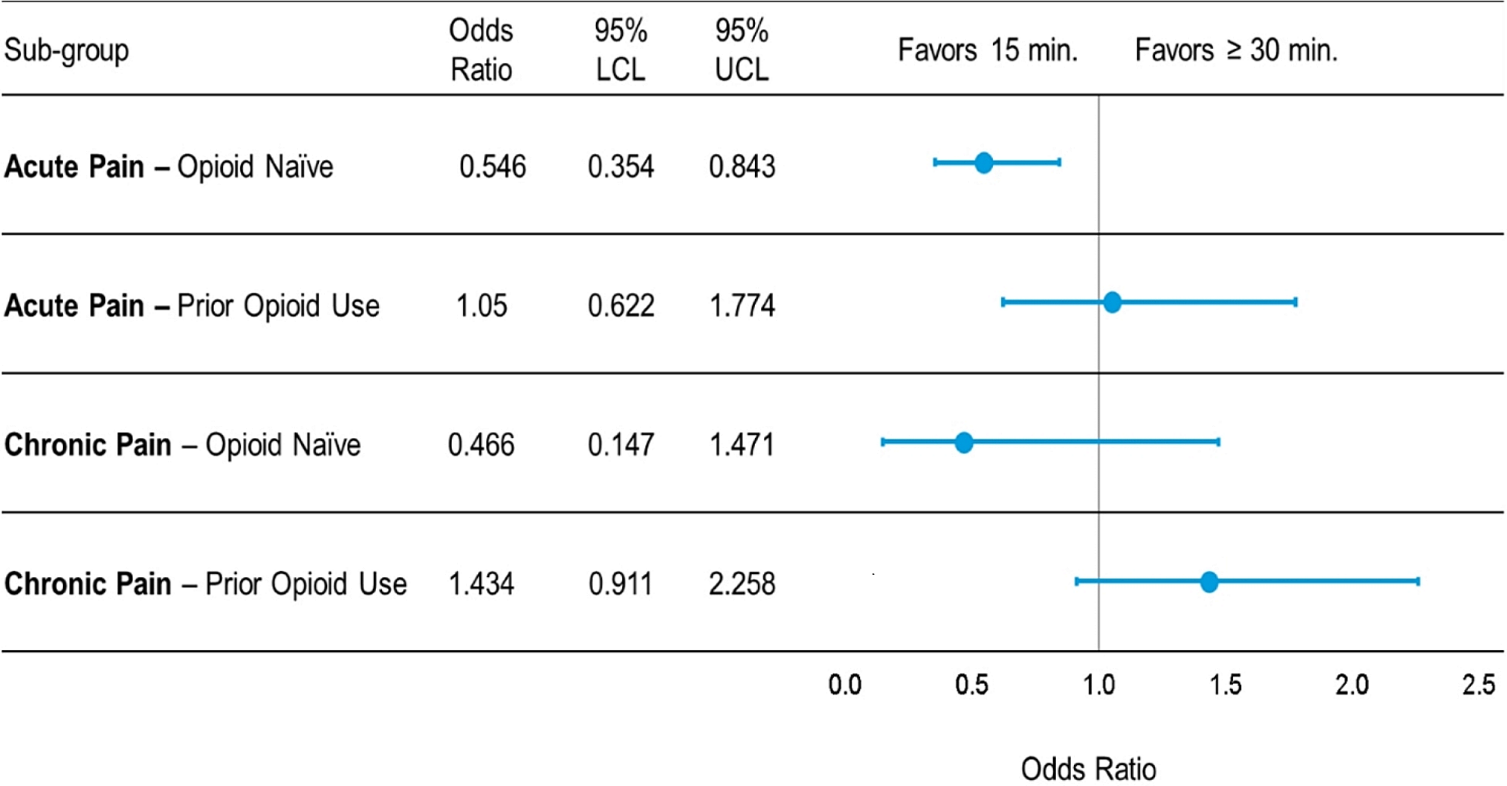
Associations between primary care appointment length and opioid prescribing in opioid-naïve and non-opioid naïve patients seen for acute and chronic pain. Four separate multivariable models examined the association between 15 minute vs. ≥30 minute scheduled primary care appointments in the four clinical scenarios of interest, adjusted for age, sex, race, ethnicity, language, marital status, practice area, provider type and Charlson Comorbidity index

Crude rates of opioid prescribing for opioid naïve patients presenting with acute pain was significantly lower for patients seen in scheduled 15-minute appointments when compared to appointments scheduled for 30 min of longer (OR 0.55, 95% CI 0.35–0.84). There were no significant differences in opioid prescribing among the other three subgroups **(**Fig. 1**)**. The full multi-variable models assessing associations between appointment lengths and opioid prescribing in each of the four subgroups are shown in Supplemental Tables 2–5.

## Discussion

Brief, 15-min primary care appointments for acute and chronic painful health conditions, as implemented across five primary care clinics within an integrated healthcare delivery system in the U.S. Upper Midwest, did not increase the likelihood of opioid prescribing. Among opioid-naïve patients presenting for evaluation of an acute pain syndrome, brief duration appointment length was associated with significantly lower odds of opioid initiation compared to longer appointments. There was no difference in the odds of opioid prescribing for patients with chronic pain (whether opioid-naïve or not) or for patients already on opioid therapy. These findings underscore the prevailing caution around opioid prescribing and hesitation to initiate therapy for new episodes of pain in patients not already receiving opioid analgesia. These findings lend important insights into the impact of limited time spent with patients and time available for shared decision-making about pain management options and opioid prescribing.

Our work builds on emerging literature suggesting that organizational and practice management variables within primary care may contribute to opioid prescribing practices. Neprash et al. [16] and Philpot et al. [17] have studied the association between time of the day and opioid prescription patterns, identifying that rates of opioid prescriptions increased later in the clinic day. Other clinician and system factors impacting opioid prescribing have also been identified [15], including lack of comfort in discontinuing previously prescribed opioids, lack of control over opioids prescribed by other clinicians outside of primary care, and limited access to comprehensive pain management programs. However, to our knowledge, this is the first study to examine the association between scheduled appointment length and rates of opioid prescribing. Similarly, while much has been written about the relationship between time pressures on physicians, burnout, and the quality-of-care provided [31, 32], and some published on the impact of time constraints on resource utilization in primary care practice [33], there was little evidence about the relationship between appointment duration and the plausible impact on the quality of clinical care provided, such as opioid prescribing.

The scheduling template utilized by our healthcare system prompts the scheduling of brief (15-minute) appointments for a limited number of painful conditions (Appendix B), which explains both the small number of 15-minute appointments and the lower odds of opioid prescribing observed during these encounters. Specifically, patients with underlying medical complexity as well as patients presenting for the evaluation of most musculoskeletal conditions are preferentially scheduled for longer duration appointments. Despite efforts to minimize confounding by patient factors that permit shorter appointment scheduling and select the subset of patients scheduled for 30-minute appointments who would be just as likely to have been scheduled for 15-minute appointments, it is likely that residual unmeasured confounding remained. These confounding variables may have made it more likely for patients to receive opioids, for example history of adverse childhood experiences, other addictions, concurrent mental health, or social determinants which impact ability to access comprehensive pain management all could have influenced these results. However, it is also possible that clinicians simply do not have the time to discuss the risks and benefits of different pain management strategies during 15-minute appointments. As a result, clinicians may be less likely to prescribe opioids to patients that require prolonged shared decision making. In contrast, the decision of whether to prescribe opioids to patients previously treated with opioids, or those with chronic pain for whom other treatment modalities haven’t worked, may not be viewed by clinicians with the same degree of hesitation, resulting in comparable prescribing rates during 15-minute and 30-minute appointment slots.

Our study is strengthened by the development and implementation of a novel, robust classification scheme for acute and chronic pain conditions. This approach, which can be implemented in different EHR and claims data sources, can help support a wide range of research and practice improvement efforts seeking to improve pain management. This is particularly important, as there is limited data on chronic pain syndromes and on acute pain overall, with most research narrowly focused on select acute or chronic pain conditions rather than overall pain management. Similarly, the internal scheduling data leveraged for this study, with clear classifications of appointment durations allows for an intuitive and reliable estimate of clinician time allotted to the patient visit in evaluating this and other important health service outcomes. duration and other important health service outcomes.

However, this work has important limitations. It is a retrospective cohort study and as such cannot establish causal effect, particularly as appointment lengths are not randomly assigned. While there was sufficient variation in scheduling to support the conducted analyses and despite propensity score methods being employed to minimize bias, there remains risk of residual confounding by factors associated with both appointment length designation and probability of being prescribed an opioid. We did not assess pain scores or other measures of care quality in this population, as such we cannot comment on appropriateness of therapy provided in these visits. The study population was limited to patients receiving primary care in one of five sites in Mayo Clinic Rochester, such that our findings may not generalize to different patient populations and settings, particularly practices seeing higher volumes of patients and those in an RVU incentivized model where brief duration appointments are more common. Practices not utilizing a centralized scheduling model may have different approaches for triaging and scheduling patients presenting with painful conditions which also may impact opioid prescribing differently.

We could not capture opioids prescribed outside of office visits, and it is possible that brief appointments led to prescriptions being issued after the visit; however, anecdotally this does not appear to be a common practice within our institution. Pain specialists and other sub-specialists do not typically initiate or manage opioids for this primary care population.

As the study of factors influencing opiate prescribing in the primary care setting remains nascent, additional research is needed to understand how time pressures and office visit dynamics, clinical support, activities such as review of prescription drug monitoring programs, primary care team composition, and patient factors influence opioid prescribing for both acute and chronic pain conditions. While there have been investigations of the association between scheduled appointment length and healthcare utilization [31] other outcomes of health care delivery also need to be examined, including clinician and patient experience, guideline concordant care delivery, quality of patient communication, and coordination of care.

## Conclusions

Understanding how the scheduled length of a primary care appointment impacts opioid prescriptions issued for both acute and chronic painful conditions is informative for clinicians, practice administrators, public health and patient safety experts, and regulatory agencies. We found that under the circumstances being considered, brief duration appointments are associated with lower risk of opioid prescription compared with longer duration appointment lengths in opioid-naïve patients presenting with acute pain episodes. In patients with prior history of opioid use, as well as opioid-naïve patients presenting for evaluation of chronic pain, scheduled appointment length had no impact on the odds of opioid prescribing. These findings can be used to improve healthcare delivery models and triage processes to provide safer care.

### Electronic supplementary material

Below is the link to the electronic supplementary material.

Supplementary Material 1

Supplementary Material 2

Supplementary Material 3

Supplementary Material 4

Supplementary Material 5

Supplementary Material 6

Supplementary Material 7

Supplementary Material 8

## Data Availability

All data generated or analyzed in this article are included in the published article and/or in the supplementary files.

## Abbreviations

ICD: International Classification of Diseases
EHR: Electronic Health Record
PCP: Primary Care Provider
MN: Minnesota
STROBE: Strengthening the reporting of observational studies in epidemiology
ED: Emergency Department
RVU: Relative Value Unit
