# Supplementary material for: The association between primary care appointment lengths and opioid prescribing for common pain conditions
Source: BMC Health Serv Res. 2024 Jul 2;24:776. doi: 10.1186/s12913-024-11215-5 (PMC11220962; doi:10.1186/s12913-024-11215-5)
Supplement: Supplementary file 1 — Supplementary Material 1 [file 12913_2024_11215_MOESM1_ESM.docx]

**Appendix B: Mayo Clinic Primary Care Acute visit scheduling template**

|  | **Patient age 18-64 years** | **Patient age ≥65 years** |
| --- | --- | --- |
| **15 minutes** | Dermatologic concerns, eye concerns, ear concerns, gastrointestinal concerns, respiratory concerns (cough [not with Asthma or COPD], sinusitis, allergies), STD check, UTI | Dermatologic concerns, eye concerns, ear concerns, gastrointestinal concerns, respiratory concerns (sinusitis, allergies), STD check, UTI |
| **30 minutes** | Mental health, edema, ED or hospital follow-up, fatigue, fainting, dizziness, gynecological/obstetrical concerns, medication checks, obesity, multiple symptoms, Pain, Pre-Op, respiratory concerns (with Asthma/COPD/shortness of breath), Sleep, orthopedic | Same as 18-64 plus cough/upper respiratory infection with or without Asthma/COPD/shortness of breath |
| **45 minutes** | New patient to primary care, Interpreter required, Physical exam with 3 or more chronic conditions (18-64), All physical exams 65 or older | |
