# Supplementary material for: The association between primary care appointment lengths and opioid prescribing for common pain conditions
Source: BMC Health Serv Res. 2024 Jul 2;24:776. doi: 10.1186/s12913-024-11215-5 (PMC11220962; doi:10.1186/s12913-024-11215-5)
Supplement: Supplementary file 3 — Supplementary Material 3 [file 12913_2024_11215_MOESM3_ESM.docx]

**Technical Appendix – Propensity Score Matching**

A variety of factors were used to build a propensity score model with the purpose of addressing the anticipated selection bias in patients received 15 versus 30 minute or longer appointments. Propensity scores were estimated using a logistic regression model predicting whether the patient was likely to receive a shorter (15-minute) or longer (30 minute and longer) appointment length. Factors used in the propensity score model to predict appointment length selection included age, practice area in which the appointment was scheduled, provider type, the language spoken by the patient, the clinic site, the severity weighted Charlson comorbidity index, and disease indication (i.e., primary diagnosis for the appointment). Interaction terms between the provider and clinic site, patient age and disease indication, patient age and practice area, patient age and provider type, as well as between patient age and clinic site were also used in the propensity score model. These interaction terms helped account for varying compositions of patients and providers within certain practice areas or clinics and, thus, varying likelihood of receiving a shorter or longer appointment.

Propensity score models, as defined above, were estimated for each of the four study subgroups (acute pain – opioid naïve, acute pain – prior opioid use, chronic pain – opioid naïve, chronic pain – prior opioid use). The distributions of the propensity scores across exposure groups (i.e., 15-minute versus 30-minute or longer) were examined for each of the four models to check for adequate overlap to ensure that a quality propensity score model was created. The overlap of the logit of the propensity scores for each study subgroup is shown in Figure A1

**Figure A1: Distribution of the logit of the propensity score across exposure groups (15 min. versus ≥ 30 min.)**


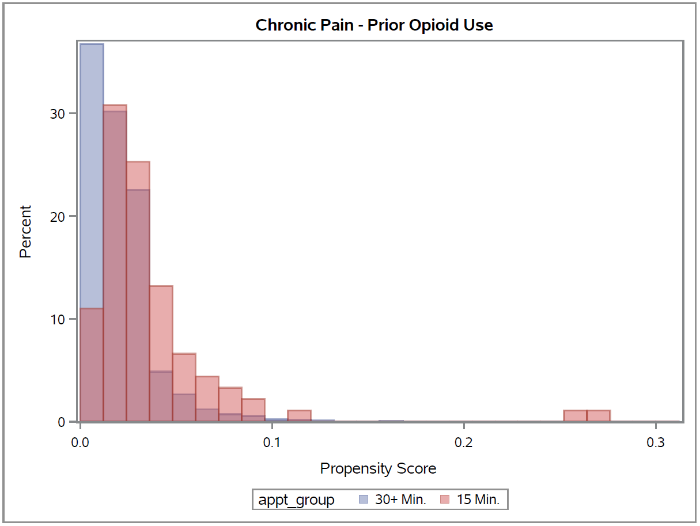

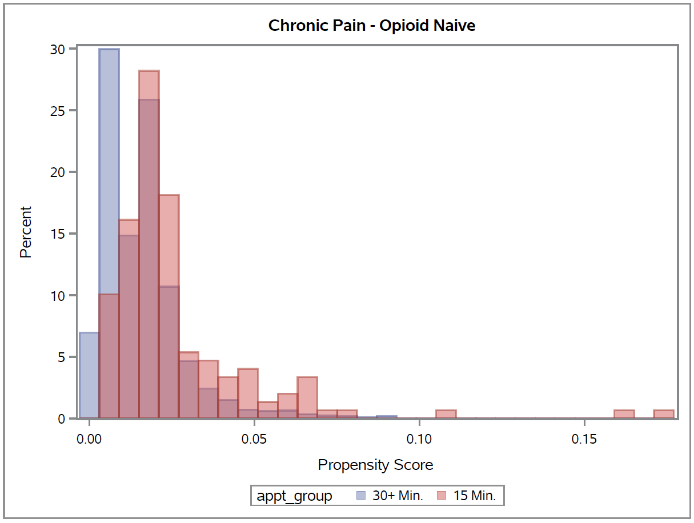

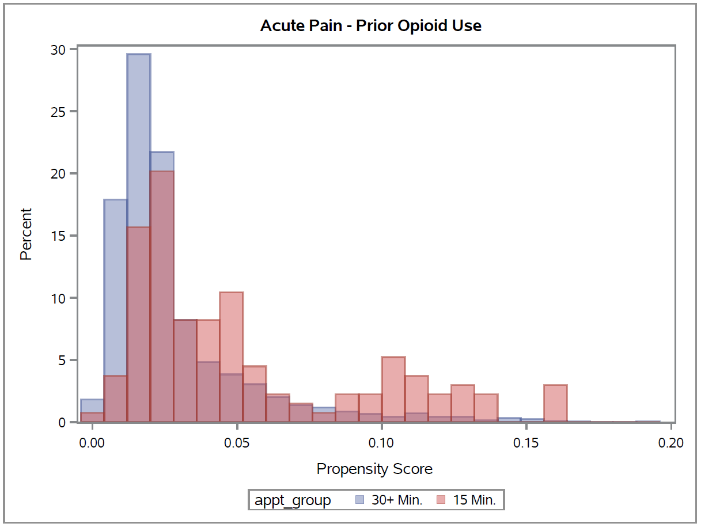

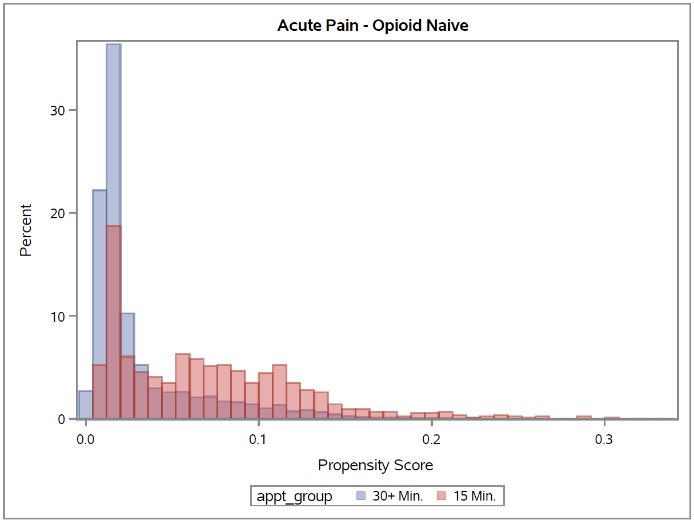


A covariate adjustment approach using the propensity score was utilized to examine the effects of the scheduled appointment length on the outcome of interest. In other words, the resulting propensity score from the propensity score model was included as a covariate in the outcome model to adjust for the likelihood of a subject receiving a 15-minute versus 30-minute or longer appointment.

1. Austin PC. An Introduction to Propensity Score Methods for Reducing the Effects of Confounding in Observational Studies. Multivariate Behav Res. 2011;46(3):399-424.

2. D'Agostino RB Jr. Propensity score methods for bias reduction in the comparison of a treatment to a non-randomized control group. Stat Med. 1998 Oct 15;17(19):2265-81.

3. Rosenbaum PR, Rubin DB. The central role of the propensity score in observational studies for causal effects. Biometrika. 1983 April; 70(1): 41-55.
