# Supplementary material for: The association between primary care appointment lengths and opioid prescribing for common pain conditions
Source: BMC Health Serv Res. 2024 Jul 2;24:776. doi: 10.1186/s12913-024-11215-5 (PMC11220962; doi:10.1186/s12913-024-11215-5)
Supplement: Supplementary file 8 — Supplementary Material 8 [file 12913_2024_11215_MOESM8_ESM.docx]

**Supplemental Table 1: Appointment length distribution by subgroup**

| **Patient Characteristics: Acute vs. chronic Pain and Opioid Naïve vs. Chronic Opioid exposure** | | **15 Minutes Appt**  **N= 1,233 (2.6%)** | **30 Minutes or Longer**  **N = 45,380 (97.4%** |
| --- | --- | --- | --- |
| **Acute Pain indication, Opioid Naïve**  **Acute Pain indication, Chronic Opioid exposure**  **Chronic Pain indication, Opioid Naïve**  **Chronic Pain indication, Chronic Opioid exposure** | | 859 (3.1%)    134 (2.8%)    149 (1.6%)    91 (2.1%) | 27,091 (96.9%)    4,702 (97.2%)     9,272 (98.4%)     4,315 (97.9%) |
|  |  |  |  |
